# Excimer Laser Coronary Atherectomy: Current Evidence, Clinical Applications, and Future Directions

**DOI:** 10.3390/jcm15020766

**Published:** 2026-01-17

**Authors:** Mohsen Mohandes, Alberto Pernigotti, Mauricio Torres, Cristina Moreno Ambroj, Francisco Fernández, Roberto Bejarano-Arosemena, Pablo Moreno, Anna Vidal-Romero, Jordi Guarinos, Jose Luis Ferreiro

**Affiliations:** Interventional Cardiology Unit, Cardiology Division, Joan XXIII University Hospital, Pere Virgili Health Research Institute (IISPV), 43005 Tarragona, Spain; mohandesmohsen@hotmail.com (M.M.); a.pernigotti@gmail.com (A.P.); maoto11@gmail.com (M.T.); crystynama@hotmail.com (C.M.A.); pacofdezmex@gmail.com (F.F.); robejarano_a@hotmail.com (R.B.-A.); moreno.pena.pablo@gmail.com (P.M.); annaaavr@gmail.com (A.V.-R.); jguarinos.hj23.ics@gencat.cat (J.G.)

**Keywords:** ELCA (excimer laser coronary atherectomy), PCI (percutaneous coronary intervention), HTB (high thrombus burden), ISR (in-stent restenosis)

## Abstract

Excimer Laser Coronary Atherectomy (ELCA) has re-emerged as a valuable adjunctive modality in percutaneous coronary intervention (PCI), particularly in the context of increasingly complex coronary anatomy and rising procedural expectations. By delivering pulsed ultraviolet energy at 308 nm through flexible fiber-optic catheters, ELCA enables precise photochemical, photothermal, and photomechanical ablation of atherosclerotic, fibrotic, calcified, and thrombotic tissue while minimizing thermal injury to surrounding structures. Recent technical refinements, simplified catheter designs, and improved safety profiles have enhanced its feasibility and utility across a range of challenging lesion subsets. This review summarizes the fundamental principles underlying excimer laser–tissue interaction, discusses available equipment and key procedural considerations, and examines the expanding clinical evidence supporting ELCA in contemporary practice. Data from observational studies and multicenter registries suggest that ELCA may enhance device crossability, restore coronary flow, and reduce distal embolization in thrombus-rich lesions, particularly during primary PCI. In device-uncrossable lesions, ELCA facilitates plaque modification and improves procedural success, including in chronic total occlusions. Furthermore, ELCA—especially when performed with simultaneous contrast injection—has demonstrated efficacy in treating stent underexpansion refractory to high-pressure balloon dilation, improving minimal stent area and enabling optimal post-dilatation. As lesion complexity continues to increase, ELCA is gaining recognition as an important tool within the interventional armamentarium. While generally safe in experienced hands, ELCA carries a risk of procedural complications that must be carefully considered. Ongoing investigations are expected to further define its optimal use and reinforce its relevance in modern interventional cardiology.

## 1. Introduction

Percutaneous coronary intervention (PCI) is increasingly performed in patients with complex coronary anatomy, in whom adjunctive plaque modification or debulking techniques may be required to achieve optimal procedural and clinical outcomes. The first clinical use of laser energy in humans was reported in 1983, when an argon laser was used to salvage an ischemic limb [1].

The application of laser technology in interventional cardiology dates back to the 1980s. First-generation laser devices emitted continuous-wave energy, which was associated with excessive coronary wall heating—often exceeding 160 °C—and an increased risk of vessel perforation [2,3].

Subsequent generations introduced pulsed-wave ultraviolet emission, enabling more precise ablation of atherosclerotic and thrombotic material with minimal thermal injury and negligible damage to adjacent tissues [4]. These technological advances have expanded the role of laser therapy in complex PCI, establishing ELCA as a valuable adjunctive technique for coronary revascularization.

Excimer Laser Coronary Atherectomy (ELCA™ Coronary Laser Atherectomy Catheter; Philips, San Diego, CA, USA) uses a xenon–chloride (XeCl) excimer laser to generate ultraviolet pulses transmitted via optical fibers to the target lesion. This review provides a summary of the current evidence, clinical applications, and future perspectives of ELCA in contemporary interventional cardiology.

## 2. Mechanism of Action and Physical Principles

**Figure 1:**
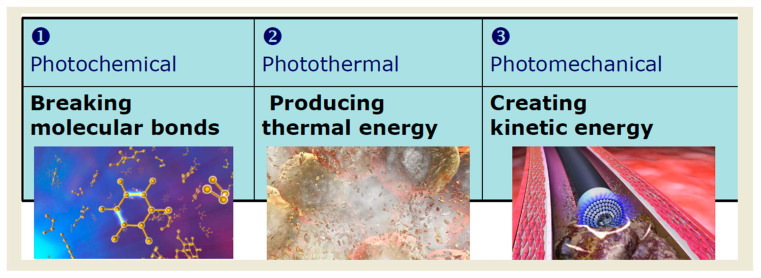
Three principal mechanisms of tissue ablation induced by the coronary excimer laser (Reprinted/adapted with permission from Philips (“© 2025 Philips”)).

ELCA uses a xenon–chloride (XeCl) excimer—an excited dimer that exists only in an energized state—as the gaseous medium to generate ultraviolet laser pulses at a wavelength of 308 nm, delivered to the target lesion through flexible optical fibers [5]. Tissue ablation occurs through three principal mechanisms:

1. Photochemical ablation: Ultraviolet photons are absorbed by atheromatous material, leading to rupture of carbon–carbon bonds and direct photodissociation of plaque components. Each laser pulse lasts approximately 120 ns, preventing significant heat transfer to surrounding tissue.
2. Photothermal effect: Molecular bond rupture increases intracellular water temperature, resulting in cell disruption and vapor bubble formation. This effect occurs over a very short duration (approximately 100 µs), thereby minimizing thermal injury to adjacent tissue.
3. Photomechanical effect: Rapid expansion and subsequent implosion of vapor bubbles further disrupt plaque and facilitate removal of ablation by-products such as water, gas, and microparticles. Most particles are <10 µm in diameter and are subsequently cleared by the reticuloendothelial system, minimizing the risk of distal embolization [6,7] (Figure 1).

## 3. Laser Equipment

**Figure 2:**
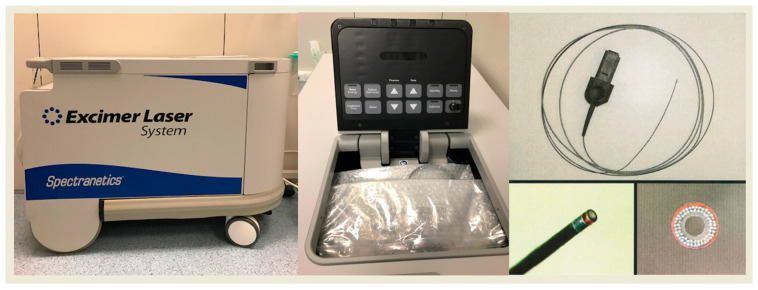
CV 300 generator and fiber optic monorail catheter.

The system consists of an excimer laser generator, most commonly the CVX-300 unit (Philips, San Diego, CA, USA), which is the predominant platform in centers where ELCA is available. This portable unit weighs 295 kg and measures 89 cm in height, 124 cm in length, and 61 cm in width (Figure 2).

Within the laser console, a high-energy electrical discharge is applied to a pressurized mixture of xenon (Xe), chlorine (Cl_2_), and a buffer gas (typically helium or neon). This excitation transiently forms the excited dimer XeCl, which is an unstable complex existing only in its excited state. As XeCl returns to its ground state, it rapidly dissociates while emitting ultraviolet photons at a wavelength of 308 nm. The generated ultraviolet energy is then transmitted through a fiber-optic catheter to the target lesion, enabling precise photochemical, photothermal, and photomechanical ablation of atherosclerotic plaque.

**Figure 3:**
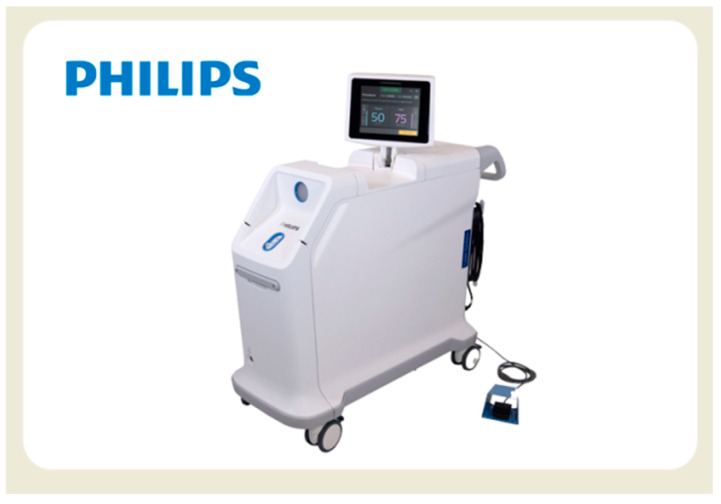
The latest laser generator system, featuring a more compact design (Reprinted/adapted with permission from Philips (“© 2025 Philips”)).

The generator requires a 5-min warm-up period before connecting the fiber-optic catheter and initiating laser application. The latest version of the generator, available in some centers, is lighter (217.7 kg) and requires only a 30-s warm-up period (Figure 3).

The laser energy is delivered through a fiber-optic catheter with an output fluence ranging from 30 to 80 mJ/mm^2^, a repetition rate of 25–80 Hz, and a pulse duration of 125–200 ns.

Laser energy delivery can be modulated by adjusting fluence (mJ/mm^2^) and pulse repetition rate (Hz), allowing tailoring of ablation intensity to lesion characteristics. Lower fluence and repetition rates are typically used during initial catheter advancement or in highly stenotic, thrombotic, or fragile lesions to minimize vessel injury. Progressive escalation of energy settings may be employed once catheter passage is achieved, particularly in fibrotic or calcified lesions. At equivalent fluence and repetition rates, larger catheter diameters deliver greater total energy per pulse and therefore generally require a more cautious stepwise escalation compared with smaller catheters.

Two main catheter designs are available: concentric and eccentric. The concentric catheter, used in most cases and typically in a monorail configuration, delivers laser energy symmetrically along the catheter’s central axis, making it suitable for standard lesion ablation. In contrast, the eccentric catheter emits the laser beam laterally, allowing more selective ablation and is particularly recommended for in-stent restenosis (ISR) and bifurcation lesions. All laser catheters incorporate a distal radiopaque marker to enable precise fluoroscopic localization of the catheter tip during the procedure. Catheters are available in different diameters—0.9, 1.4, 1.7, and 2.0 mm—and are compatible with all standard 0.014-inch guidewires [8,9]. The rapid exchange concentric coronary catheters come in 0.9 mm, 1.4 mm, 1.7 mm, and 2.0 mm while the eccentric catheter measure in 1.7 and 2 mm in size. The latest concentric catheters feature improved spacing of optical fibers, resulting in a wider exit angle and an increased effective ablation area. Importantly, catheter size selection is primarily guided by lesion morphology and degree of luminal compromise rather than reference vessel diameter, in contrast to balloon angioplasty. In severely stenotic or uncrossable lesions, smaller-diameter catheters are generally preferred to facilitate lesion crossing and allow safe, stepwise energy escalation [10].

Catheter compatibility with guiding catheters varies by size according to manufacturer specifications. The 0.9 mm catheter is designed for use through a 6 Fr guiding catheter, the 1.4 mm catheter through 6–7 Fr, the 1.7 mm catheter through 7 Fr, and the 2.0 mm catheter through 8 Fr. These recommendations help optimize deliverability and support during ELCA procedures (Table 1).

## 4. Procedure

After completion of the generator warm-up, the laser catheter is connected to the console and calibrated to ensure accurate energy delivery. The system delivers pulsed ultraviolet energy in synchrony with saline infusion, which is essential to prevent coronary wall heating and optimize ablation efficiency.

Laser energy is delivered in an on–off sequence, typically consisting of 10 s of laser activation followed by 5 s of rest for the 0.9 mm catheter, and 5 s of activation followed by 10 s of rest for larger catheter sizes (1.4 mm, 1.7 mm, 2.0 mm). An audible alert marks the end of each rest period, at which point the next lasing cycle can be initiated. This intermittent delivery protocol helps prevent thermal injury, minimize gas accumulation, and maintain stable hemodynamics during ablation [5].

**Figure 4:**
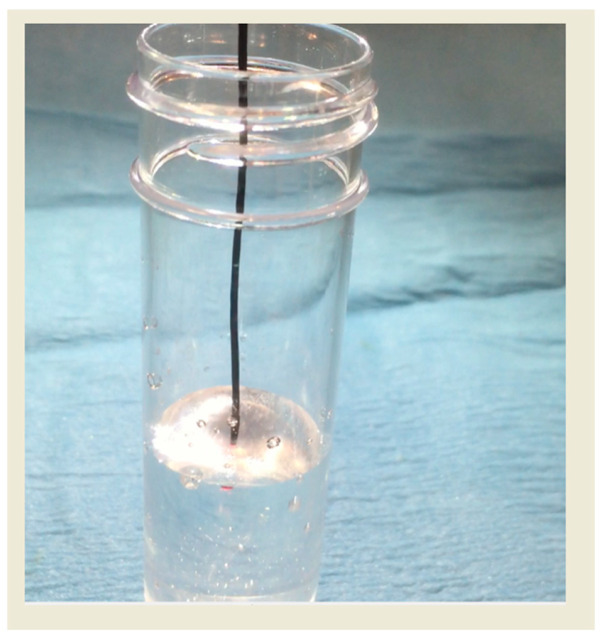
Interaction of the laser with contrast media generates microbubble.

After successful guidewire crossing and before laser activation, contrast medium and blood must be thoroughly flushed from the coronary artery using saline. This step is critical because both contrast and blood contain non-aqueous macromolecules, such as proteins, that can absorb a substantial proportion of laser energy. Such absorption promotes microbubble formation, with rapid expansion and collapse generating transient intraluminal pressures of up to approximately 100 atmospheres, potentially leading to coronary dissection or perforation. Adequate saline flushing ensures a clear optical path and minimizes the risk of these explosive vaporization effects during laser delivery [11,12] (Figure 4).

In routine practice, a 1 L bag of 0.9% normal saline is connected to the guiding catheter via a three-way stopcock, and saline infusion is adjusted to approximately 2–3 mL/s during each laser application. The laser catheter should be advanced very slowly—at a rate of approximately 0.5 mm/s—through the target lesion, given the limited penetration depth of the laser (35–50 µm) [10]. This careful technique optimizes laser–tissue interaction and protects against thermal injury. In our practice, an additional saline bolus of approximately 3–4 mL is administered through the guiding catheter during the rest period to further reduce the risk of excessive heating.

Importantly, the objective of ELCA is not mechanical lesion penetration in a “Dotter-like” fashion, but rather controlled delivery of laser energy to achieve true photoablation of the target tissue. A major advantage of ELCA is that the laser catheter can be advanced over any standard 0.014-inch guidewire, in contrast to other atherectomy techniques that require dedicated guidewires [13].

## 5. Indications and Clinical Evidence

### 5.1. Thrombotic Lesion

Patients presenting with a high thrombus burden (HTB), particularly in the setting of primary PCI, represent a major challenge in contemporary interventional cardiology. Manual thrombus aspiration (MTA) has traditionally been used to reduce intracoronary thrombus burden. However, unsuccessful thrombus aspiration in ST-segment elevation myocardial infarction (STEMI) is not uncommon and has been associated with impaired myocardial perfusion, increased microvascular obstruction, and adverse effects on survival [14]. The last randomized trial evaluating routine MTA in STEMI, the TOTAL trial [15] compared MTA plus PCI with PCI alone and demonstrated no benefit in reducing cardiovascular death, recurrent myocardial infarction (MI), cardiogenic shock, or New York Heart Association (NYHA) class IV heart failure at 180 days. Moreover, routine MTA was associated with an increased risk of stroke within 30 days.

Although data evaluating ELCA in thrombotic lesions—particularly during primary PCI—remain limited, available evidence suggests that coronary laser therapy may be beneficial in selected patients with HTB. Beyond its mechanical ability to fragment intracoronary thrombus into very small particles, excimer laser energy has been shown, at least in in vitro models, to alter platelet aggregation kinetics by reducing platelet aggregation and contractile force development. This so-called “stunned platelet” effect appears to be dose-dependent and is most pronounced at higher fluence levels, such as 60 mJ/mm^2^ [16].

**Figure 5:**
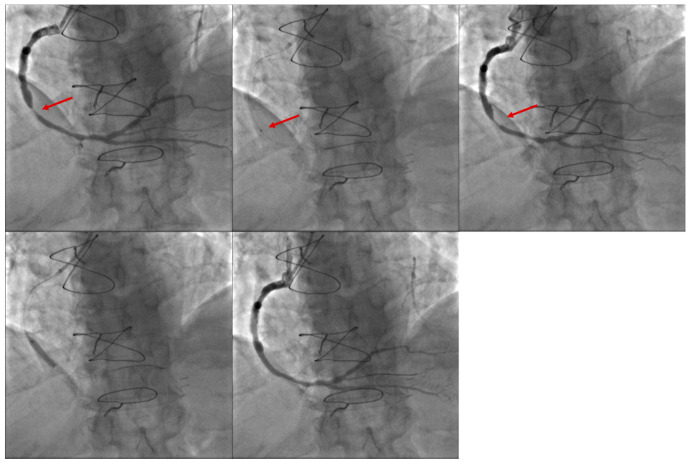
A coronary angiogram in a 69-year-old man presenting with non–ST-elevation myocardial infarction (NSTEMI) showed a severe lesion (**upper left**, arrowhead) in the saphenous vein graft (SVG) to the right coronary artery (RCA). Excimer laser coronary atherectomy (ELCA) was performed for lesion ablation using a 0.9 mm catheter (**upper middle**, arrowhead), with 45 mJ/mm^2^ of fluence and a frequency of 25 Hz for 90 s, delivering a total of 2250 pulses. Following laser application (**upper right**, arrowhead), a direct drug-eluting stent (DES) (3.5 × 15 mm) was implanted (**bottom left**), achieving a good final angiographic result (**bottom right**).

The CARMEL multicenter trial [17] enrolled 151 patients with MI, of whom 65% presented with HTB. Following laser application, significant improvements in Thrombolysis in Myocardial Infarction (TIMI) flow grade and minimal lumen diameter were observed, with the greatest degree of thrombus reduction occurring in patients with HTB. Rates of distal embolization and no-reflow were low, at 2% and 3%, respectively. Notably, in 21% of cases the culprit lesion was located in a saphenous vein graft (SVG), a setting frequently associated with HTB (Figure 5).

In a retrospective analysis, Shishikura D et al. [18], compared ELCA (*n* = 50) with MTA (*n* = 48) in patients presenting with acute coronary syndrome (ACS), predominantly STEMI. Lesion crossability was significantly higher in the ELCA group (96.2%) than in the MTA group (82.6%). In addition, the proportion of patients achieving TIMI grade 3 flow and myocardial blush grade (MBG) 3 was significantly greater in the ELCA group, whereas ST-segment resolution did not differ between groups. It should be noted that the lower crossability in the MTA group reflects limitations of the aspiration device in highly thrombotic lesions, rather than an inability to cross the artery in routine clinical practice.

Iiya M et al. [19], conducted a retrospective study including 586 patients undergoing primary PCI, comparing outcomes between those treated with ELCA (*n* = 256) and those who did not receive ELCA (*n* = 330). The ELCA group showed a significantly lower incidence of major adverse cardiovascular events (MACE), defined as cardiac death, nonfatal myocardial infarction, and target lesion revascularization (TLR) (6% vs. 15% in the ELCA and non-ELCA groups, respectively). This difference was mainly driven by a reduced rate of TLR in the ELCA group. Rates of coronary perforation and distal embolization were low and did not differ significantly between the two groups.

Arai T et al. [20], retrospectively evaluated patients with ACS presenting with TIMI 0 flow on initial angiography and compared outcomes between an ELCA group (*n* = 48) and MTA group (*n* = 50). The ELCA group demonstrated shorter door-to-reperfusion times (89.2 ± 6.7 vs. 137.9 ± 12.3 min, respectively), lower peak creatine kinase–myocardial band (CK-MB) levels (242 ± 25 vs. 384 ± 63 IU/L), improved myocardial blush grade, and a more favorable in-hospital MACE profile compared with the MTA group (8% vs. 20%, respectively).

Similarly, Shimojo K et al. [21] analyzed 143 patients with STEMI treated with either ELCA (*n* = 63) or MTA (*n* = 80). Patients treated with ELCA exhibited significantly lower peak CK-MB levels (190.0 [70.5–342.0] IU/L in the ELCA group vs. 256.5 [157.0–354.8] IU/L in the MTA group) and greater improvement in left ventricular ejection fraction at three months (14.1 [6.2–19.8]% vs. 9.5 [3.9–15.3]%, respectively), as assessed by single-photon emission computed tomography (SPECT).

**Figure 6:**
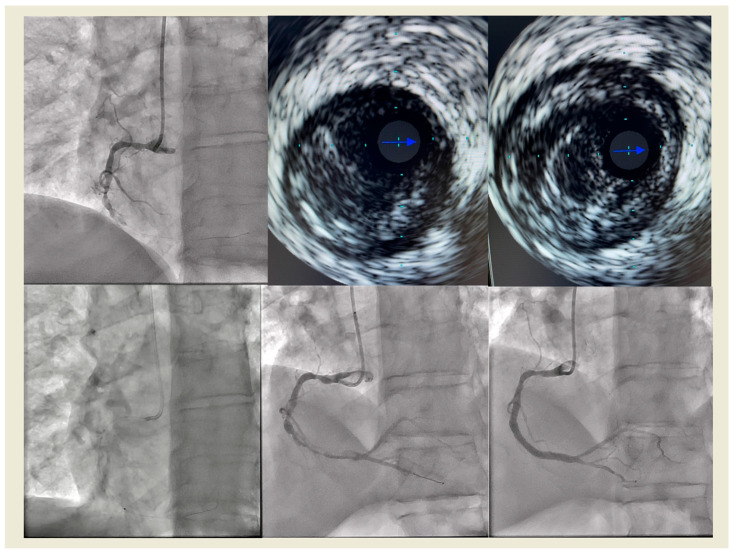
Right coronary artery (RCA) with a high thrombus burden (HTB) in a patient with STEMI (**upper left**). After wire crossing, coronary flow was not restored, revealing substantial thrombus within an aneurysmal vessel, as demonstrated by IVUS (**upper middle** and **upper right**). Following several passes of ELCA (**lower left**), coronary flow was restored (**lower middle**). Three drug-eluting (DES) stents were then directly implanted, achieving a good angiographic result with TIMI III flow (**lower right**).

We recently reported our experience with ELCA as an adjunctive therapy during primary PCI in 130 patients with STEMI, 95.4% of whom presented with HTB. Technical and procedural success were achieved in 98.5% and 95.4% of cases, respectively. Rates of distal embolization (2.3%) and no-reflow (3.1%) were low, with only one case of coronary perforation reported [22] (Figure 6).

Finally, Yamanaka Y et al. [23] evaluated the thrombus vaporization effect of ELCA using optical coherence tomography (OCT) in 92 patients with STEMI. After ELCA was performed following MTA, residual thrombus volume was significantly reduced, from 65.7 mm^3^ after MTA to 47.5 mm^3^ after ELCA. Plaque rupture as the underlying pathological substrate was identified by OCT in only 22 cases (23.9%) after MTA but became distinguishable in an additional 36 cases after ELCA (total of 58 cases; 63.0%). Moreover, ruptured lesions exhibited significantly greater thrombus reduction with ELCA (21.2 mm^3^ vs. 11.8 mm^3^).

In addition to mechanical thrombus management with MTA or ELCA, pharmacological strategies are commonly employed in HTB lesions in patients with ACS undergoing PCI. Glycoprotein IIb/IIIa inhibitors may be used adjunctively to reduce platelet aggregation and facilitate thrombus resolution, particularly in patients with HTB or suboptimal flow. Importantly, the use of GP IIb/IIIa inhibitors is compatible with ELCA, and concurrent administration does not interfere with laser application. While routine use has declined in many centers due to bleeding risk and the availability of potent oral P2Y12 inhibitors, GP IIb/IIIa inhibition remains an important tool in selected high-risk cases, such as patients presenting with HTB, no-reflow, or slow flow, where its use aims to improve procedural success and reduce infarct size [24].

Overall, the available evidence suggests that ELCA may represent a useful adjunctive option in selected patients with a HTB, particularly, during primary PCI for STEMI. Observational studies and intravascular imaging data indicate that ELCA can potentially improve epicardial flow, microvascular perfusion, without an apparent increase in major procedural complications. Compared with MTA, ELCA may offer advantages in terms of lesion crossability and thrombus removal, especially in highly thrombotic or resistant lesions. In the absence of large randomized trials, ELCA may be considered as a selective or bailout strategy rather than a routine approach, particularly in cases of HTB and or inadequate thrombus removal after MTA. Further prospective randomized studies are warranted to better define its clinical role and optimal patient selection.

### 5.2. Device Uncrossable Lesion

**Figure 7:**
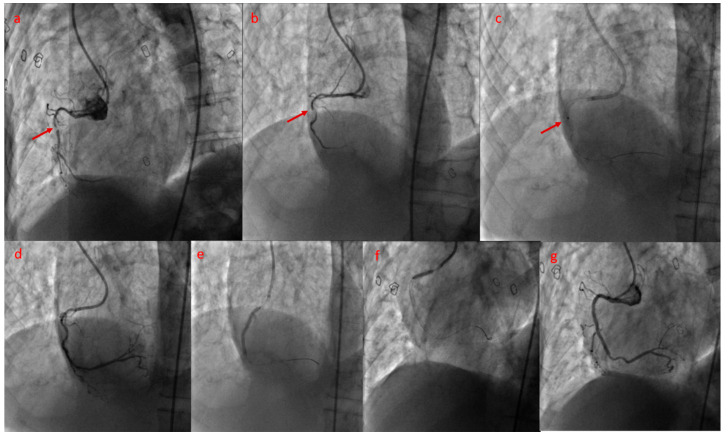
Subtotal occlusion of the mid segment of the right coronary artery (RCA) with a calcified component ((**a**), arrowhead) in a 71-year-old man, which behaved as uncrossable to microcatheter advancement (Caravel; Asahi Intecc, Aichi, Japan) after a Pilot 50 wire (Abbott Vascular, Santa Clara, CA, USA) traversed the lesion ((**b**), arrowhead). Excimer laser coronary atherectomy (ELCA) was performed with a 0.9 mm catheter ((**c**), arrowhead), applying maximum fluence and frequency settings (80 mJ/mm^2^ and 80 Hz, respectively) for a total of 40 s and 2800 pulses, achieving adequate ablation of the lesion (**d**). Subsequently, the microcatheter crossed the lesion and the Pilot 50 wire was exchanged for a Sion wire. After multiple balloon predilations, two drug-eluting stents (DES) (2.5 × 38 mm and 2.75 × 12 mm; (**e**,**f**)) were implanted in the mid and proximal RCA, obtaining a good final angiographic result (**g**).

In certain situations, after successful lesion crossing with a guidewire, no device is able to traverse the lesion. In the setting of chronic total occlusion (CTO), device-uncrossable lesions occur in up to 9% of cases and represent the second most frequent cause of procedural failure, after failure to cross the lesion with a guidewire [25,26]. A major advantage of ELCA in this scenario is that the laser catheter can be advanced over the existing guidewire to ablate the proximal cap and facilitate subsequent device passage (Figure 7). Although the laser catheter may not cross the lesion in all cases, it can modify and debulk the proximal cap, thereby facilitating lesion dilation and increasing procedural success in more than 80% of cases in our experience [27]. Karacsonyi J [28] in a study of 4845 CTO lesions, identified 752 lesions (15.5%) as either balloon-uncrossable (523 cases) or balloon-undilatable (356 cases). The study reported significantly higher technical (91.5% vs. 83.1%) and procedural success rates (88.9% vs. 81.6%) in the ELCA-treated group compared with those in whom the technique was not employed, with comparable complication rates (3.92% vs. 3.51%). Ojeda S et al. [29] in a multicenter registry including 126 uncrossable lesions in 126 patients, reported that technical and procedural success were achieved in 114 (90.5%) and 110 (87.3%) patients, respectively. Rotational atherectomy was attempted as a bailout strategy in 21 of 23 ELCA failures (91.3%) and was successful in 14 cases (66.7%). Severe lesion calcification was independently associated with ELCA failure.

Litvack F et al. [30] in an analysis of 3000 patients undergoing PCI with ELCA, including 10% with CTO, reported laser and procedural success rates of 84% and 89%, respectively. Notably, although complication rates varied among lesion types, CTOs and vein grafts exhibited the lowest complication rates. The study did not specify whether these CTOs were uncrossable lesions.

It is worth noting that CTO-PCI represents a complex scenario, with uncrossable lesions adding an additional layer of procedural difficulty and potential risk. Sintek M et al. [31] in an analysis of ELCA safety using the NCDR/CATH PCI Registry, including various indications for laser use, reported a significantly higher rate of a combined endpoint—death, tamponade, any dissection, or any perforation—in CTOs where ELCA was employed compared with 16,812 CTOs without ELCA use (6.7% vs. 3.7%, respectively). While ELCA can facilitate the crossing and modification of these challenging lesions, careful procedural planning and operator experience are essential to minimize potential complications.

### 5.3. Stent Underexpansion

Fibrotic and calcified lesions often require adequate lesion preparation prior to stent implantation, as the minimal stent area (MSA) assessed by intravascular ultrasound (IVUS) is a consistent predictor of ISR [32,33]. Stent underexpansion and residual reference segment stenosis are also associated with an increased risk of stent thrombosis [34]. Optimal lesion preparation before stent deployment is therefore essential to achieve favorable long-term outcomes.

Although the ideal approach is to prevent stent underexpansion, once it occurs it poses a significant challenge. High-pressure post-dilatation with a non-compliant (NC) balloon is commonly employed; however, its efficacy is often limited. Chandrasekhar J et al. demonstrated using digital stent enhancement that adequate stent expansion increased only from 21% to 58% following NC balloon post-dilatation [35].

ELCA with simultaneous contrast injection is an off-label technique used to manage this complication. The concurrent injection of contrast generates microbubbles (Figure 4), which can ablate the surrounding resistant tissue contributing to stent underexpansion. This facilitates further expansion with an NC balloon—even in cases where high-pressure NC balloon post-dilatation was previously ineffective [6,36].

In our experience with 16 patients, ELCA with simultaneous contrast injection was performed to address stent underexpansion. IVUS was used in all cases (except one) to measure MSA before and after the procedure, demonstrating an average increase of 2.34 ± 1.57 mm^2^ following laser ablation and subsequent NC balloon dilation [37].

In line with the aforementioned evidence supporting ELCA for the treatment of stent underexpansion, intravascular lithotripsy (IVL) has also been safely used as an adjunctive technique in this setting [38]. The multicenter IVL-ELCA Dragon Registry [39] compared IVL (69 patients) and ELCA (52 patients) in patients with stent underexpansion. Procedural success, assessed by intravascular imaging and defined as technical success with a final stent expansion ≥80%, was comparable between both techniques (IVL 76.8% vs. ELCA 75%). Notably, ELCA was associated with a greater relative increase in stent expansion from pre-PCI to post-PCI (58.0 vs. 47.6; *p* < 0.001), while no significant differences were observed in the final MSA between the two groups (5.1 vs. 5.1 mm^2^; *p* = 0.53). Both approaches were associated with a low and similar incidence of short-term adverse cardiovascular events.

### 5.4. In-Stent Restenosis

ISR remains a significant clinical challenge, primarily due to its high recurrence rate. ISR is a response to vessel wall injury that leads to excessive tissue proliferation—either neointimal hyperplasia or neoatherosclerosis—within the stented segment. It is primarily diagnosed angiographically, defined as a diameter stenosis >50% within the stent, including 5 mm proximal and distal to the stent edges. In the era of drug-eluting stents (DES), the incidence of clinically significant ISR is approximately 10% within five years after implantation [40]. Assessment of the underlying mechanism using intracoronary imaging during ISR treatment is crucial to guide therapy and minimize recurrence [41]. Both DES and drug-coated balloons (DCB) have been used for ISR treatment, with DCBs particularly attractive because they avoid implanting an additional metallic layer. However, the 2024 ESC Guidelines recommend DES implantation over DCB for DES-ISR [42] reflecting evidence from the DAEDALUS pooled analysis, which included 1976 patients from 10 randomized trials [43]. This study demonstrated that treatment with paclitaxel-coated balloons (PCB) was associated with a higher risk of TLR at 3 years compared with DES (hazard ratio [HR] 1.32, 95% CI 1.02–1.70; *p* = 0.035). Regardless of the definitive therapy, optimal lesion preparation—through tissue modification using cutting or scoring balloons, or high-pressure NC balloon dilation—remains essential to achieve improved long-term outcomes.

He P et al. [44] compared outcomes in 85 ISR lesions from 75 patients treated with ELCA plus DCB (*n* = 24) versus DCB alone (*n* = 61). The ELCA + DCB group demonstrated greater acute lumen gain (3.57 ± 0.79 mm^2^ vs. 2.50 ± 1.06 mm^2^) and a lower rate of TLR at one-year follow-up (hazard ratio 0.33, 95% CI 0.11–0.99). Additionally, they observed that ELCA with concurrent contrast injection was associated with greater acute lumen gain compared with ELCA with saline infusion.

The adjunctive use of ELCA for tissue ablation in ISR, combined with its ability to achieve greater acute lumen gain, makes it an attractive option for lesion preparation. In a retrospective analysis of 87 lesions in 81 patients with DES–ISR undergoing PCI with or without ELCA, Ichimoto E et al. [45] reported significantly greater acute lumen gain in the ELCA group compared with the non-ELCA group (1.64 ± 0.48 mm versus 1.26 ± 0.42 mm, *p* < 0.001). Long-term clinical outcomes were similar between the groups, despite the ELCA cohort having significantly more complex lesions.

The combination of ELCA with DCB angioplasty represents a particularly attractive strategy for the treatment of ISR, as it enables effective intrastent tissue debulking while avoiding the implantation of an additional metallic layer. In a randomized study including 110 patients with ISR treated with DCB angioplasty, He P et al. [46] allocated patients to ELCA followed by DCB (*n* = 55) or DCB alone (*n* = 55). Lesion preparation with ELCA was associated with a significantly lower incidence of TLR at 1 year compared with standard DCB angioplasty (hazard ratio 0.38; 95% CI 0.15–0.95; *p* = 0.038).

Regarding the underlying mechanisms and tissue characteristics of ISR, Ishihara T et al. [47] conducted a retrospective observational study of 208 ISR lesions treated with DCB under OCT guidance. The study compared acute lumen gain, measured by quantitative coronary angiography, and clinically driven (CD) TLR between the ELCA group (*n* = 47) and the no-ELCA group (*n* = 161). The authors found that ELCA use was associated with significantly greater acute lumen gain in lesions with a homogeneous pattern, although it did not have a significant impact on CD-TLR.

Moreover, in cases of ISR in which the underlying pathological substrate is stent underexpansion due to peri-stent calcium—a particularly challenging scenario—ELCA has demonstrated effectiveness in modifying calcified tissue, resulting in greater final lumen dimensions and improved expansion of the previously implanted stent [48].

ISR involving multiple metallic layers represents a particularly challenging subset, in which further stent implantation is undesirable due to the risk of increased vessel rigidity, impaired vasomotion, and recurrent restenosis. In this context, ELCA may offer specific advantages by enabling effective debulking of intrastent tissue without adding an additional metallic layer and by facilitating adequate expansion of the previously implanted stent [49].

A large retrospective analysis recently published evaluated long-term outcomes in patients undergoing ELCA-assisted PCI, including 448 of 21,256 patients (2.1%) treated with ELCA [50]. In this observational cohort, ELCA was generally used in lesions considered more complex, such as those with ISR, stent underexpansion, or multilayer stents. The overall incidence of target vessel revascularization in ELCA-treated patients was 16.7%, increasing to 29.5% in cases where the indication was ISR. These findings likely reflect the higher baseline lesion complexity in the ELCA-treated group, rather than a direct effect of the technique itself. Importantly, no independent association between ELCA and long-term mortality was observed, supporting its potential role as an adjunctive tool in selected, complex ISR lesions.

Finally, an emerging technique in the management of ISR and stent underexpansion involves the use of contrast–saline mixtures during ELCA to enhance laser energy within the stented segment. In this approach, contrast is mixed with saline, typically in ratios ranging from approximately 25% to 75% contrast, to maximize effective energy delivery when standard saline flush techniques are insufficient, particularly in complex or heavily calcified ISR lesions. Wacinski P et al. [51] in a prospective observational registry of 52 patients (73 lesions) with ISR due to underexpanded stents in which standard flush and batch ELCA techniques had failed, demonstrated that ELCA using a contrast–saline mixture facilitated an increase in average cross-sectional area assessed by IVUS/OCT from 2.9 to 7.3 mm^2^ without major vessel perforation, supporting its feasibility and safety in this setting.

These preliminary findings suggest that contrast–saline mixture ELCA may improve lesion modification and stent expansion in selected ISR cases. While the optimal mixture ratios and procedural protocols remain to be defined, this approach represents a promising adjunctive strategy for complex or resistant intrastent lesions.

## 6. Complications

ELCA is generally safe in experienced hands; however, a range of complications may occur and should be carefully considered. Major procedural complications include coronary dissection, coronary perforation, distal embolization, slow-flow/no-reflow phenomenon, ventricular arrhythmias, and procedure-related myocardial infarction. Reported complication rates vary according to lesion complexity, patient population, and operator experience.

In a large series of 3000 patients undergoing angioplasty with ELCA Litvack F et al. [30] reported rates of in-hospital bypass surgery of 3.8%, Q-wave MI of 2.1%, death of 0.5%, and coronary perforation of 1.2%. Coronary dissection has been reported in 3–5% of cases [7].

In the CARAMEL multicenter registry [17] which included 151 patients with acute MI treated with ELCA (65% with HTB and 21% with SVG as the culprit vessel), distal embolization occurred in 2% of cases. Similarly, in our own series of 130 patients with STEMI, of whom 95.4% presented with HTB, ELCA was associated with a 2.3% rate of distal embolization [22]. Ventricular tachycardia or ventricular fibrillation has been reported in approximately 1.6% of cases [29].

Certain lesion characteristics—including severe calcification, CTO, vessel tortuosity, and stent underexpansion—are potentially associated with a higher risk of adverse events. Procedural factors such as rapid advancement of the laser catheter, use of high fluence without adequate saline flushing, or inappropriate contrast injection may further increase complication risk.

Preventive strategies include careful lesion assessment with intracoronary imaging, gradual stepwise escalation of laser energy, complete blood and contrast washout, continuous saline infusion, slow catheter advancement, and appropriate patient selection. Prompt recognition and management of complications—such as balloon tamponade for perforation, intracoronary vasodilators for no-reflow, and standard interventional techniques for flow-limiting dissection—are essential to maintain procedural safety and optimize outcomes. Overall, although complications remain relatively uncommon, their recognition and proactive management are critical for the safe and effective use of ELCA.

## 7. Technical and Procedural Considerations

As discussed above, ELCA is a safe and effective technique across a wide range of clinical scenarios and is associated with a relatively low rate of procedural complications when performed appropriately. Nevertheless, strict adherence to specific technical and procedural principles is essential to maximize efficacy and ensure safety.

First, thorough blood and contrast washout prior to laser activation, along with continuous saline infusion during laser delivery, is mandatory to minimize coronary wall heating and reduce the risk of complications. This aspect is particularly important during thrombus vaporization, as blood stagnation may interact with laser energy and lead to microbubble formation, potentially increasing the risk of distal embolization or vessel injury. Simultaneous contrast injection should generally be avoided and reserved for selected cases of stent underexpansion, after intracoronary imaging (IVUS or OCT) confirms the presence of a circumferential calcified ring as the underlying mechanism.

Second, slow and controlled advancement of the laser catheter is crucial to allow adequate energy absorption by the target tissue and to avoid excessive mechanical or thermal injury. Third, the maximum laser catheter diameter should not exceed two-thirds of the reference vessel diameter to reduce the risk of dissection or perforation. Fourth, catheter size, fluence, and repetition rate should be individualized according to lesion characteristics and clinical indication.

In our practice, maximum fluence and repetition rate (80 mJ/mm^2^ and 80 Hz) using a 0.9 mm catheter are reserved for uncrossable or extremely resistant lesions, whereas low-to-moderate fluence and frequency settings are typically used for other indications, including thrombotic lesions or in-stent restenosis.

Finally, because the laser catheter is relatively rigid, special caution is required when navigating tortuous or angulated coronary segments. ELCA should be avoided in vessels with severe angulation (>60°), where the risk of vessel injury or catheter-related complications may be increased [11].

## 8. Future Perspectives

As coronary interventions continue to evolve toward the treatment of increasingly complex and high-risk lesions, the role of adjunctive technologies in facilitating and optimizing procedural outcomes is becoming increasingly important. Although observational studies and multicenter registries have consistently reported encouraging results with ELCA across a broad spectrum of challenging clinical scenarios, robust randomized controlled trials remain limited.

In particular, prospective studies specifically designed to evaluate the efficacy of ELCA for thrombus vaporization in STEMI patients with HTB, as well as its potential impact on microvascular obstruction and myocardial reperfusion, are warranted. Likewise, further evidence is needed to better define the role of ELCA in the management of in-stent restenosis, especially in comparison with contemporary lesion preparation strategies and drug-based therapies, and to determine whether ELCA confers incremental benefits on long-term clinical outcomes in this setting.

The integration of ELCA with intracoronary imaging–guided PCI may further improve procedural planning by enabling more accurate identification of lesion characteristics predictive of procedural success, reducing complication rates, and facilitating personalized treatment strategies.

Despite its potential benefits, the adoption of ELCA remains influenced by procedural complexity, operator experience, device cost, and limited availability in many healthcare systems, factors that may restrict its use to selected centers. While additional high-quality evidence is awaited, ELCA continues to occupy a distinct and valuable niche within the interventional armamentarium, particularly in thrombus-rich lesions, device-uncrossable segments, and selected cases of stent underexpansion or ISR. Careful patient selection and strict adherence to technical recommendations and procedural nuances remain essential to maximize clinical benefit while minimizing procedural risk.

## 9. Conclusions

ELCA is a safe and effective adjunctive therapy in complex PCI, particularly in thrombus-rich lesions, device-uncrossable segments, and selected cases of stent underexpansion or ISR. Evidence derived from observational studies and multicenter registries supports its high procedural success and a favorable safety profile when appropriate technical principles are applied. Although well-designed randomized controlled trials are still needed to further define its role across different clinical scenarios, ELCA remains a valuable and versatile tool within the interventional armamentarium when used with careful patient selection and meticulous procedural technique.

## Figures and Tables

**Table 1:** Practical considerations for ELCA catheter size selection and energy delivery.

Catheter Diameter	Design Available	Typical Lesion Characteristics	Relative Energy Delivery *	Practical Energy Strategy
0.9 mm	Concentric	Severely stenotic or uncrossable lesions; thrombotic lesions	Low	Initiate at low fluence and repetition rate; gradual, stepwise escalation once catheter advancement is achieved
1.4 mm	Concentric	Fibrotic or moderately calcified lesions	Moderate	Intermediate fluence with cautious escalation based on lesion response
1.7 mm	Concentric/Eccentric	In-stent restenosis, bifurcation lesions, plaque modification	Higher	Careful stepwise escalation; eccentric catheter for selective ablation when appropriate
2.0 mm	Concentric/Eccentric	Larger luminal lesions; selected cases of ISR	Highest	Conservative initial settings due to higher total energy delivery; escalation only after stable catheter positioning

* Relative energy delivery refers to total energy delivered per pulse at equivalent fluence and repetition rate.

## Data Availability

Data derived from our own experience are included in this review article. All other information supporting the findings is provided in the article. Further inquiries can be directed to the corresponding author.
